# Device-Free Human Identification Using Behavior Signatures in WiFi Sensing

**DOI:** 10.3390/s21175921

**Published:** 2021-09-03

**Authors:** Ronghui Zhang, Xiaojun Jing

**Affiliations:** School of Information and Communication Engineering, Beijing University of Posts and Telecommunications, Beijing 100876, China; zrhmaster@163.com

**Keywords:** device-free, deep learning, human identification, channel state information, wireless sensing

## Abstract

Wireless sensing can be used for human identification by mining and quantifying individual behavior effects on wireless signal propagation. This work proposes a novel device-free biometric (DFB) system, WirelessID, that explores the joint human fine-grained behavior and body physical signatures embedded in channel state information (CSI) by extracting spatiotemporal features. In addition, the signal fluctuations corresponding to different parts of the body contribute differently to the identification performance. Inspired by the success of the attention mechanism in computer vision (CV), thus, to extract more robust features, we introduce the spatiotemporal attention function into our system. To evaluate the performance, commercial WiFi devices are used for prototyping WirelessID in a real laboratory environment with an average accuracy of 93.14% and a best accuracy of 97.72% for five individuals.

## 1. Introduction

Presently, developments in wireless sensing technologies have shown that wireless signals can be deployed to transmit information between wireless communication devices and are also able to realize object wireless sensing [1]. Movements of individuals within the coverage of wireless signals will inevitably impact signal propagation. These effects on wireless signals are recorded as channel state information (CSI). The mining and quantifying of such effects in CSI without additional sensors such as cameras, radars, or wearable devices are the main focus of device-free wireless sensing (DFWS).

Biometrics or biological recognition is the automatic identification of individuals by quantifying their biological and behavioral characteristics [2]. Pioneering studies have explored the inherent influence of the human body or human behavior on wireless signal propagation to recognize individuals using commercial WiFi, which is typically referred to as device-free biometrics (DFB).

### 1.1. Motivation

Previous attempts at DFB have mainly focused on biometric features such as gait [3,4], respiration [5], and radio biometrics [6]. Although bandwidth is limited, WiFi still exhibits similar functions to those of radar in terms of indoor sensing. Yunze Zeng et al. [3] demonstrated that the gait information of an individual hidden in the CSI is sufficient for confirming his/her identity. Wei Wang et al. [4] performed time–frequency transformation on the CSI waveform to obtain a spectrogram to extract walking patterns. Jie Wang et al. [5] proposed an empirical mode-decomposition-based general DFI framework to extract intrinsic features for DFB. Differences in individual physical characteristics (such as height and weight, body water content, skin conditions, and other biological tissues) cause differential interference with wireless signal propagation [6]. Based on the above insights, Qinyi Xu et al. [6] utilized a time-reversal (TR) technique to explore the radio biometrics of different individuals for DFB.

The above works aimed to identify the unique biometric characteristics (behavioral or physical signature of individuals) hidden in CSI. The objectives of these works are either to discover the characteristics of the coarse-grained behavior itself or to discover the characteristics of the physical characteristics of the human body without considering them as a single entity. Practically, when a person makes a gesture, it will inevitably lead to the movement of other parts of the body, which together with the stationary part of the body causes a disturbance in wireless signal propagation. Motivated by the above insight, in this work, we propose a novel DFB system, WirelessID, that explores the joint human fine-grained behavior and body physical signatures embedded in CSI by extracting spatiotemporal features. In addition, the signal fluctuations corresponding to different parts of the body contribute differently to identification performance. That is, different parts of a CSI sample and different CSI series contribute differently to the features. Inspired by the success of the attention mechanism [7] in computer vision (CV), thus, to extract more robust features, we introduce the spatiotemporal attention function into our deep model that automatically assigns weight according to its importance for performance improvement.

### 1.2. Contributions

The main contributions of this work are as follows:

- We leverage, for the first time, the joint human fine-grained behavior and body physical signature embedded in CSI for human identification;
- We propose a novel DFB system, WirelessID. To evaluate the performance, commercial WiFi devices are used for prototyping WirelessID in a real laboratory environment. The recognition rate of the test has an average accuracy of 93.14% and a best accuracy of 97.72% for five individuals.

### 1.3. Organization

The remaining structure of this work is organized as follows. We introduce the related work in Section 2. In Section 3, the system architecture of WirelessID is elaborated, focusing on two modules: sensing signal acquisition and preprocessing; spatiotemporal feature extraction. This is followed by experiments on the performance evaluation of WirelessID with a discussion, given in Section 4. We conclude this work in Section 6.

## 2. Related Work

### 2.1. Human Identification

Human identification is the basis of various applications, such as smart homes and security. In existing systems, cameras and radars are commonly used sensors for identity recognition. The static features hidden in fingerprint [8], iris [9], and face [10] images are mined and used for identity recognition, while radars or cameras capture the dynamic characteristics of the human body in the gait for identification [11,12,13]. The high cost limits the popularity of radar systems in daily life. Ordinary cameras are inexpensive and easy to deploy, but have a high risk of privacy leakage. Compared with the sensing techniques mentioned above, wireless sensing does not require special sensor equipment, can control privacy disclosure to a low level, can function normally in smoky or dark environments, and represents important technical support for achieving ubiquitous sensing [1]. Currently, researchers from industry and academia are actively promoting wireless sensing technologies for human identification [3,4,5,6,14]. Inspired by their positive results, this work explores identity recognition by mining the unique patterns of individuals hidden in wireless sensing signals.

### 2.2. Device-Free Wireless Sensing for Human Detection

WiFi signals contain plentiful information, such as time of arrival (ToA), angle of arrival (AoA), and CSI, that can be used to achieve various functions similar to radar systems [15]. The achievements in this field are roughly divided into model-based methods (such as the Fresnel model) and data-driven/pattern-based methods (such as deep learning), which we introduce respectively below.

#### 2.2.1. Model-Based Methods for DFWS

By mapping the relationship between signal fluctuations and human activities in the area surrounded by wireless signals, the model-based method realizes DFWS [16]. The Fresnel zone model was introduced into DFWS to characterize the properties of wireless signal propagation, thereby realizing respiration detection [17]. CARM proposed two models: the CSI-speed model and the CSI-activity model for human activity recognition by modeling the relationships among the frequencies of CSI power variations, the human movement speeds, and a specific human activity [18]. Model-based methods have been successful in some specific application scenarios with special designs. With the success of deep learning in computer vision (CV), ubiquitous sensing methods are expected to be realized. Data-driven DFWS is becoming a research hotspot.

#### 2.2.2. Data-Driven Methods for DFWS

Deep neural networks were originally designed to handle image classification and recognition problems in CV. For this, X. Wang et al. transformed AoAs estimations into images to train a DCNN for indoor localization [19]. CsiGAN was proposed to solve the classification problem of categories not included in the training set by using the generative adversarial network (GAN) to generate diverse fake samples [20]. Another WiFi sensing problem is that when people walk out of the best sensing area, the sensing performance will drop sharply. To solve this problem, F. Wang [21] proposed to construct multiple separated antenna pairs to enhance spatial diversity. The above works are to improve the ability of WiFi sensing from the perspective of information sensing and data enhancement and further to extract highly distinguishable features by deep models.

### 2.3. Attention Model

Human visual attention was studied by Rensink [22] in 2000. Ten years later, it was introduced into CV by Hinton et al. [23] and Denil et al. [24]. Since then, the attention mechanism has been widely used in CV and proven to be successful in various applications, such as video description [25,26], activity recognition [27], and object recognition [28]. Humans exploit a sequence of partial glimpses and selectively focus on the salient parts to capture the visual structure better [28]. F. Wang et al. [29] incorporated an attention mechanism with a CNN to obtain attention-aware features for improving the image classification performance. Instead of deeply embedding the attention map operation in the CNN model, CBAM [28] built a convolutional block attention module that can cooperate with any existing CNN architecture in a plug-and-play manner. CBAM learned spatial and channel-wise features by exploiting the interspatial relationship of features and the interchannel relationship of features, respectively. By exploiting the intertemporal relationship of features, the attention mechanism was extended to the temporal domain [30,31]. For example, Bengio et al. [30] achieved attention allocation by the weighted sum of the intermediate outputs of an RNN for machine translation.

These attention models learn to select the most relevant part of the data for the task implicitly. Inspired by them, this work explores the use of the spatiotemporal attention mechanism in DFWS to refine the spatiotemporal features and improve recognition performance.

## 3. WirelessID

**Figure 1:**
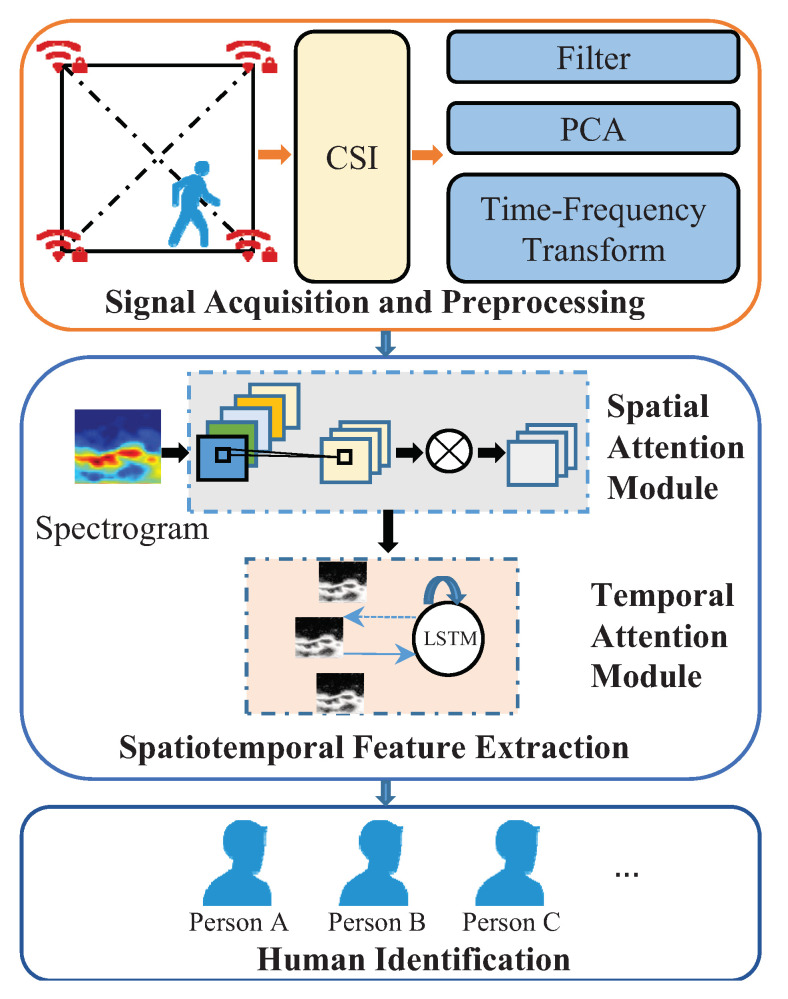
Architecture of Wireless ID.

As shown in Figure 1, the device-free human identification process of WirelessID mainly contains three stages: (1) sensing signal acquisition and preprocessing; (2) spatiotemporal feature extraction; (3) human identification. The details of each part are presented below.

### 3.1. Sensing Signal Acquisition and Preprocessing

Currently, wireless channels can be measured by commercial WiFi devices. x(t) and y(t) represent the transmitted and received signals at time *t*, respectively. CSI can be expressed as H(f,t)=Y(f,t)/X(f,t), where X(f,t) and Y(f,t) are frequency domain representations of x(t) and y(t), respectively [18].

Taking into account the multipath effects on the wireless signal in the sensing area, CSI can be formulated as follows [32]:

(1) $$H\left(f,t\right)=\left(\sum_{n=1}^{N}\alpha_{n}\left(f,t\right)e^{-j2\pi f\tau_{n}\left(f,t\right)}\right)e^{j\varepsilon \left(f,t\right)},$$

where *N* indicates the total number of paths, $\alpha_{n}\left(f,t\right)$ and $\tau_{n}\left(f,t\right)$ are the complex attenuation and propagation delay of the $n^{th}$ path, respectively, and $e^{j\epsilon \left(f,t\right)}$ is the phase shift caused by timing alignment offset, sampling frequency offset, and carrier frequency offset.

To reveal the Doppler frequency shift (DFS), which is similar to what is observed in Doppler radar results [4], a transformation of CSI is formulated as follows [32]:

(2) $$\begin{array}{c} H\left(f,t\right)=\left(H_{s}\left(f\right)+\sum_{n\in P_{d}}\alpha_{n}\left(t\right)e^{j2\pi \int_{-\infty }^{t}f{}_{D_{n}}\left(u\right)du}\right)\times e^{j\varepsilon \left(f,t\right)}, \end{array}$$

where $H_{s}\left(f\right)$ is the sum of CSI for all static paths (without DFS) and $P_{d}$ is the set of dynamic paths caused by target movements (with DFS).

Due to the imperfections of commercial WiFi devices, the raw CSI data are always noisy. The signal fluctuations caused by human behavior are submerged in noise. As the signal fluctuations in the OFDM subcarriers are correlated, we used a principal component analysis (PCA)-based denoising algorithm [18] before a further denoising process through conjugate multiplication of the CSI of two antennas [32,33]. We performed a short-term Fourier transform (STFT) on the denoised CSI data to obtain DFS [18]. Nonzero DFS is caused by human fine-grained behaviors (including human gestures and such movements introduced by other parts of the body). Only the spectrograms of nonzero DFS were then used for spatiotemporal feature extraction.

### 3.2. Spatiotemporal Feature Extraction

Wireless signals are inherently deficient in spatial resolution, which means that all signal fluctuations caused by human behavior are difficult to capture and record in the CSI. This problem can be solved to a certain extent by using multiple antennas and multiple subcarriers. However, the sensing data are usually high-dimensional. The capability of deep learning (DL) to automatically learn forceful features at multiple levels of abstraction, rather than relying entirely on artificially constructed features, is becoming increasingly important with the continuing growth in the data size [34]. Based on the above insights, we chose DL for CSI feature extraction. To learn more robust features for improving identification performance, our feature extraction module contains two submodules: an attention-spatial module and an attention-temporal module, which obtain spatial features and temporal features by the convolutional neural networks and long short-term memory model, respectively. The details of the CNN and LSTM with the attention approach are presented below.

#### 3.2.1. Attention-Spatial Model

Multiwireless link sensing enhances spatial resolution, and the spatial information is hidden in the high-dimensional CSI data. By performing the convolution operation (operated by multiple filters) of CNN [1] on a spectrogram, spatial features can be obtained. Additionally, different frequency components and other signatures in the spectrogram contribute differently to the maximization of recognition performance. In other words, certain frequency components play a major role in recognition. An attention mechanism has been used in object tracking and recognition [24], which learns to select images to minimize tracking uncertainty. Applying pooling and convolution operations has been shown to be effective to generate a spatial attention map [28,35]. Thus, as in Equation (3), we utilized average-pooling and max-pooling on *F*, generating two 2D maps. We then concatenated these two maps. This was followed by a convolution operation and a sigmoid function to produce a spatial attention map, as Equation (4). Given an intermediate feature map *F*∈R${}^{C\times H\times W}$, the spatial attention is the degree of attention to different positions on the feature map. Mathematically, as in Equation (4), it means that for *F*, effective spatial attention corresponds to a matrix S(F)∈R${}^{H\times W}$, each position of which is a weight for the pixel at the corresponding position of *F* by performing elementwise multiplication.

(3) $$\begin{array}{cc} S(F) & =Sig\left(Conv\left(\left[AvgPool\left(F\right);MaxPool\left(F\right)\right]\right)\right), \end{array}$$

(4) $$F_{s}=S\left(F\right)\cdot F,$$

where $\left[\cdot \right]$ represents concatenating average-pooled features with max-pooled features, Conv is the convolution operation, Sig represents the sigmoid function, and · denotes elementwise multiplication between the spatial attention and the input feature map.

Therefore, we applied the operation as detailed in Equation (4) to extract spatial attention features in our spectrogram. The output of this model is input to the attention-temporal model to learn temporal attention features. The next section details the attention-temporal model.

#### 3.2.2. Attention-Temporal Model

The spectrum sequence contains the dynamics of complete behavior over time. Spectrogram sequences related to behavior may have different lengths because performing different behaviors may take different amounts of time, and different users exhibiting the same behavior may take different amounts of time. We used LSTM to encode the temporal dynamic information of a sequence. Particularly, LSTM with an attention mechanism preserves the intermediate encoding sequence results and then adaptively models a subset of these intermediate output results [30]. In other words, as shown in Equation (5), the model automatically assigns different weights to the learned features *f* according to the importance of the final recognition performance improvement. A softmax function is used to evaluate the importance of feature $f_{i}$ and outputs a regularized score $a_{i}$. As a result, as shown in Equation (6), the temporal attention features are obtained by performing multiplication of the learned features and their scores.

(5) $$a_{i}=Softmax\left(W^{T}f_{i}+b\right),$$

(6) $$F_{t}=\sum_{n=1}^{N}a_{i}*f_{i},$$

where *W* denotes the weight vector and *b* denotes the bias.

### 3.3. Human Identification

Human identification is a typical multiclass classification process, so softmax was selected as our activation function [36]. The features extracted from the above step were used to train a softmax classifier. The output of the classifier represents the probability distribution of the five human identities.

## 4. Experiment and Evaluation

### 4.1. Experiment Setup

**Figure 2:**
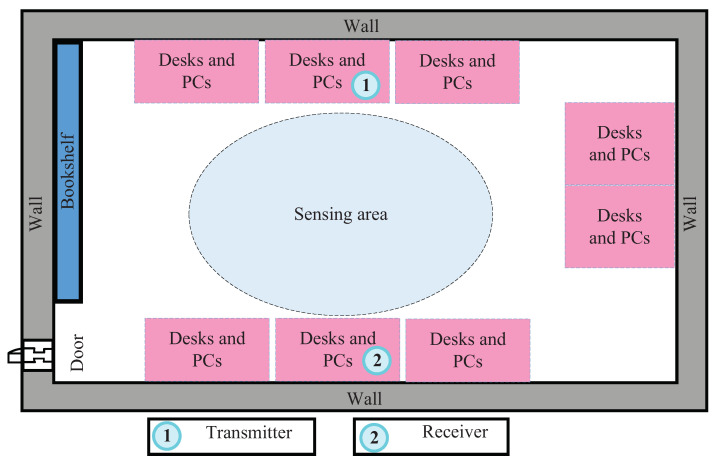
Experimental deployment scenario.

We conducted CSI measurements with the Linux 802.11n CSI Tool [1] on commercial WiFi cards deployed in a laboratory. As shown in Figure 2, the laboratory was occupied by eight sets of tables and chairs. A computer with one antenna was deployed to transmit signals and to enhance the sensing signal spatial resolution, and six antennas were deployed on another computer to receive the signals. Five users of different heights and weights (details in Table 1) successively performed three gestures (drawing Arabic numerals 1, 2, and 3) between the transmitting and receiving antennas. The obtained sensing data were saved as CSI for further processing (as described in Section 3.1) with a sampling rate of 1000, to obtain a DFS spectrogram. The number of spectrogram samples for each class was 1200–1500, 70% of which were randomly selected as the training set and the remaining 30% of which were selected as the test set. We ensured that no test data were used for the training process.

All experiments were conducted on a TensorFlow 1.8 platform deployed on a server running Ubuntu 16.04 LTS with one RTX2080Ti-11G GPU. In the training phase, training data batches were input into the deep model continuously until the model converged with a learning rate of 0.001 and a batch size of 128. The test data were then used to test the model performance.

### 4.2. Performance Evaluation

We constructed various DL network models (CNN with attention, LSTM with attention, CNN-LSTM, and CNN-LSTM with attention) and verified the impact of the number of antennas on their identification rate. The network architectures of the CNN and LSTM with the spatiotemporal attention used in this work are shown in Table 2. The impact of the amount of training data on the performance was also tested. Based on the above experiments, we compared the top-1 accuracy of the models. In particular, we experimented with WirelessID’s cross-gesture identification performance to guide the implementation of the system in real life. Furthermore, we compared the performance with the baselines.

#### 4.2.1. Impact of the Number of Receiving Antennas

**Figure 3:**
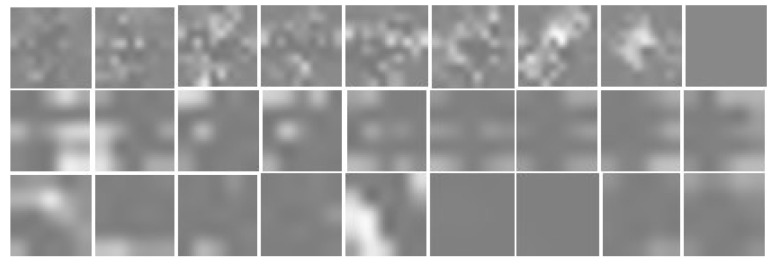
Output features of the middle layer of the deep model.

**Figure 4:**
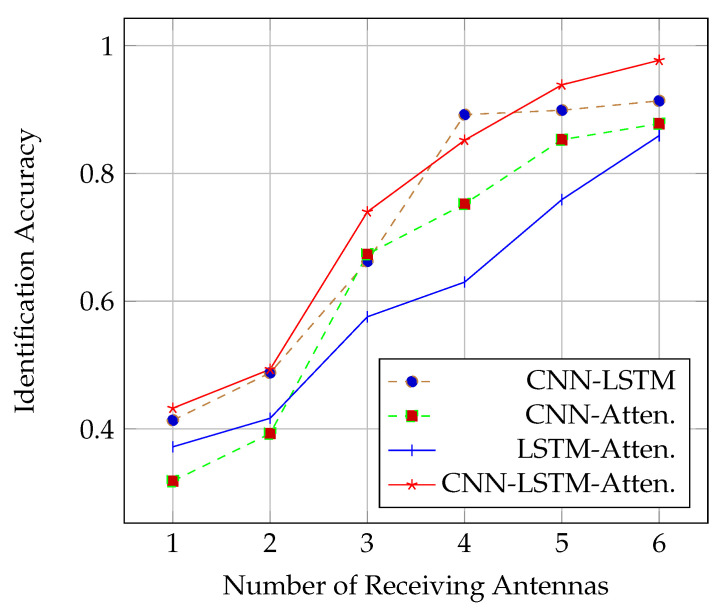
Identification rate in terms of the number of receiving antennas.

As maintained above, the fine-grained gesture and the movement of other parts of the body cause a disturbance in wireless signal propagation. We designed a deep model to obtain the personalized features of different users hidden in the signal fluctuations. We first visualize the personalized features of the middle layer of the deep model that were used to distinguish different users as Figure 3. We then studied how the performance of the DL network models varies with the number of receiving antennas. As shown in Figure 4, in almost all cases (except for that with 4 antennas), the CNN-LSTM with attention approach performed the best. In addition, the performance of all deep models experienced a significant improvement when the number of receiving antennas increased from 2 to 3, but it slowly improved when it increased from 3 to 6. The worst performance occurred in the case of a single receiving antenna. The main reason is that a single link is not enough to capture sufficient spatial characteristics to distinguish different users. Since the best performance of all deep models appeared in the case of 6 antennas, our subsequent experiments were based on 6 antennas.

#### 4.2.2. Impact of the Usage Percentage of the Training Set

**Figure 5:**
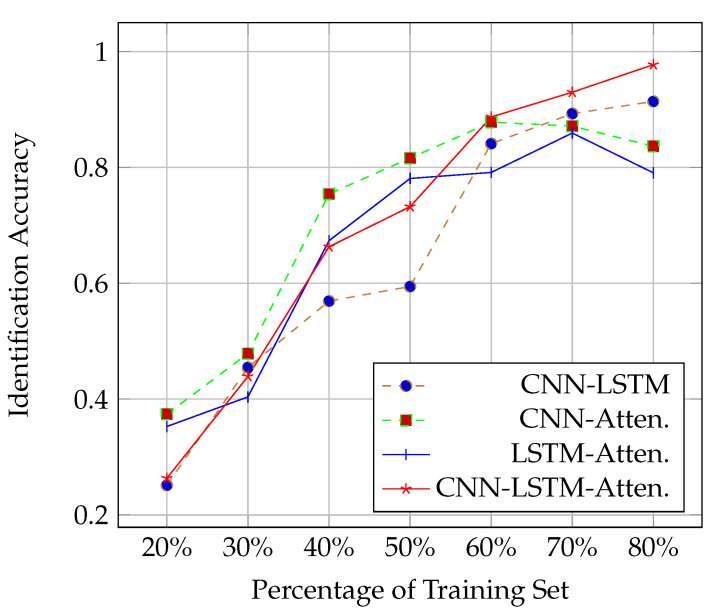
Identification rate in terms of the usage percentage of the training set.

This section presents the performance of the DL network models when the usage percentage of the training set was varied using 6 antennas and keeping the network structure unchanged according to Section 4.2.1. As shown in Figure 5, in the initial stage, due to insufficient training data, all the deep models overfit, resulting in poor performance. As the amount of training data increased, CNN with attention achieved the best performance (using 60% of the training set) and, later, LSTM with attention (using 70% of the training set). The most likely reason is that the two models are simpler than the other two and do not require too much data to converge. Considering the economic cost of data, the model trained on 80% of our training set is already acceptable.

#### 4.2.3. Comparison of the Deep Models

We compare the performance of different deep model structures in this section, especially the impact of the attention mechanism using 80% of the training set. The result is shown in Table 3. The top-1 accuracy of CNN-LSTM with attention was about 7% higher than that of CNN-LSTM, indicating the effectiveness of the attention mechanism. However, the performance of the CNN or LSTM with attention approach was not as good as that of the CNN-LSTM without attention approach, which illustrates the importance of spatiotemporal characteristics for identity recognition.

#### 4.2.4. Cross-Behavior Performance Evaluation

**Figure 6:**
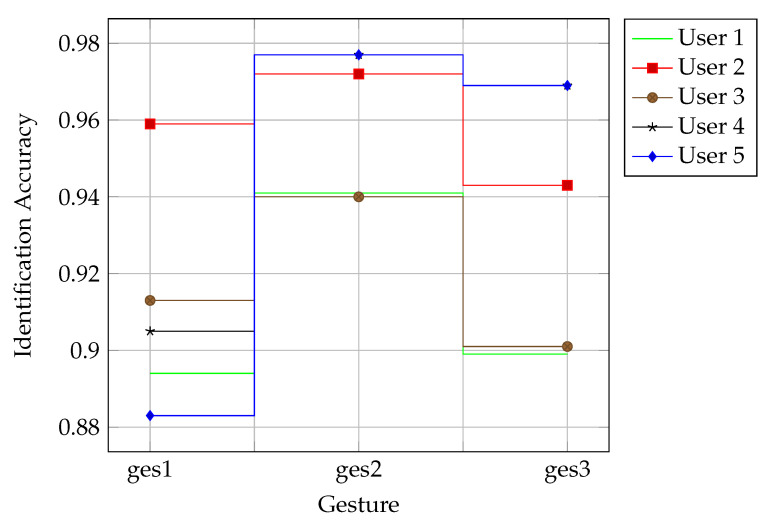
Cross-behavior identification rate of WirelessID.

The performance of WirelessID was evaluated under different behaviors with the purpose of testing whether the performance is independent of behavior by using 6 antennas and 80% of the training set. Only training data containing one gesture were used to train and test the model at a time. Therefore, the same experiment was conducted three times. As shown in Figure 6, the experimental results demonstrated that WirelessID had robust identification performance, with an average accuracy of 93.14% for five users across three gestures. The best accuracy was 97.72% for User 5 with the tallest height and heaviest weight. The accuracy of Gesture 1 was typically lower than that of the other two gestures. The most likely reason is that Gesture 1 (drawing Arabic numeral 1) was too simple, and the signal fluctuation caused by it was not rich enough to distinguish between identities well. The above insights indicated that the performance of WirelessID depends on gestures to a certain extent, of which moderately complex gestures are more suitable.

## 5. Comparisons with the Baselines

We compared the performance with that of WiID [37], which is the first gesture-based human identification work. WiID utilized the motion contour of body parts as the power-based feature for user identification while we leveraged the nonzero DFS only caused by human fine-grained behaviors (including human gestures and such movements introduced by other parts of the body). WiID achieved an average accuracy of 91.8% in the lab. The internal environment of their lab was simpler than ours (complex environment affects sensing performance). Our work achieved an average accuracy of 93.14%, which is better than that of WiID. Note that the best accuracy of our work could be up to 97.72%, showing that by the careful design of gestures, our model can meet the application standards of the real world.

## 6. Conclusions

In this work, we leveraged, for the first time, the joint human fine-grained behavior and body physical signature embedded in CSI for human identification. Signal fluctuations corresponding to different parts of the body contribute differently to identification performance. To extract more robust features, we introduced an attention mechanism into our deep spatiotemporal model. To evaluate the performance, commercial WiFi devices were used for prototyping WirelessID in a real laboratory environment. We tested the impact of receiving antenna numbers and the impact of the usage percentage of the training set. We also compared the performance of different deep models, and the cross-behavior performance evaluation demonstrated that WirelessID had an average accuracy of 93.14% and a best accuracy of 97.72% for five individuals. Note that our experiment was conducted in a stable environment, that is only the behavior of the user and the surrounding static objects affected the signal propagation at the experimental site. Regarding the influence of unstable wireless signals on activity sensing, Giuseppe Bianchi et al. performed a sufficient analysis, the details of which can be found in [38].

## Figures and Tables

**Table 1:** User information.

Users	Gender	Height (cm)	Weight (kg)
1	Female	155	45
2	Female	160	59
3	Male	164	56
4	Male	176	62
5	Male	181	75

**Table 2:** Network architectures of the CNN and LSTM with spatiotemporal attention.

No.	Operation	Configuration
1	Input	121 × 30 × 6
2	Conv1	Kernel = 3 × 3, Stride = [1, 1, 1, 1]
3	Activation	ReLU
4	Pooling	Max pooling, ksize = [1, 2, 2, 1], Strides = [1, 2, 2, 1]
5	Conv2	Kernel = 5 × 5, Stride = [1, 1, 1, 1]
6	Activation	ReLU
7	Conv3	Kernel = 5 × 5, Stride = [1, 1, 1, 1]
8	Activation	ReLU
9	Spatial Attention	Max pooling = [1, 2, 2, 1], Average pooling = [1, 2, 2, 1], Concatenate, Conv (Kernel = 5 × 5, Stride = [1, 1, 1, 1]), Sigmoid
10	Multiplication	Element-wise multiplication
11	LSTM	Input_size = 1500, Hidden_size = 128, Output_size = 128, Num_layers = 5
12	Temporal Attention	Attention_vec = 1 × 128
13	Multiplication	Dot product
14	Dense	Softmax

**Table 3:** Performance comparison of deep models.

Method	CNN-LSTM	CNN with Attention	LSTM with Attention	CNN-LSTM with Attention
Top-1 accuracy	0.9137	0.8779	0.8592	0.9772

## Data Availability

Not applicable.
